# Abdominal cocoon syndrome (idiopathic sclerosing encapsulating peritonitis): An extremely rare cause of small bowel obstruction—Two case reports and a review of literature

**DOI:** 10.3389/fmed.2022.1003775

**Published:** 2022-10-12

**Authors:** Humood A. Alsadery, Saleh Busbait, Abdulrahman AlBlowi, Morshed Alsawidan, Hassan Mohammed AlBisher, Shadi Alshammary

**Affiliations:** Department of General Surgery, College of Medicine, Imam Abdurahman Bin Faisal University, Al-Khobar, Saudi Arabia

**Keywords:** primary, idiopathic, intestinal obstruction, sclerosing encapsulation peritonitis, abdominal cocoon, peritoneal encapsulation syndrome

## Abstract

**Introduction:**

Sclerosing encapsulating peritonitis (SEP) is a rare cause of intestinal obstruction in which the bowel and internal abdominal organs are wrapped with a fibrocollagenous cocoon-like encapsulating membrane [1,2]. SEP is divided into two entities: abdominal cocoons (AC), also known as idiopathic or primary sclerosing encapsulating peritonitis, which is of extremely rare type, and secondary sclerosing encapsulating peritonitis, which is the more common type.

**Case presentation:**

Two male patients from India, a 26 year old and a 36 year old, presented to our hospital complaining about abdominal pain associated with nausea and vomiting without any history of previous surgical interventions; the patients' vitals were stable. Preoperative diagnosis of abdominal cocoon was established by abdominal computed tomography. It showed multiple dilated fluid-filled small bowel loops in the center of the abdominal cavity with thin soft tissue, non-enhancing capsules encasing the small bowel loops with mesenteric congestion involving small and large bowel loops. Both patients underwent complete surgical excision of the sac without intraoperative complications. Patients had a smooth postoperative hospital course and were discharged home in good conditions.

**Conclusion:**

Patients with abdominal cocoons have a non-specific clinical presentation of intestinal obstruction. A high index of clinical suspicion in combination with the appropriate radiological investigation will increase the chance of preoperative detection of the abdominal cocoon. In patients with complete bowel obstruction, complete excision of the peritoneal sac is the standard of care.

## Introduction

Sclerosing encapsulating peritonitis (SEP) is a rare cause of intestinal obstruction in which the bowel and internal abdominal organs are wrapped with a fibrocollagenous cocoon-like encapsulating membrane (1, 2). SEP is divided into two entities: abdominal cocoon (AC), also known as idiopathic or primary sclerosing encapsulating peritonitis, which is of extremely rare type, and secondary sclerosing encapsulating peritonitis, which is the more common type (1, 2). AC clinical manifestations are usually non-specific and most of them diagnosed intraoperatively with a thick fibrotic membrane encasing the small bowel (3). Abdominal cocoon terminology was first used by Foo et al. in 1978; however, the condition was initially described by Owtschinnikow in 1907 as peritonitis chronica fibrosa incapsulata (4, 5). Primary sclerosing encapsulating peritonitis (SEP) has no known cause or associated condition; however, secondary SEP had a similar clinical presentation to AC in association with a clear high-risk condition like abdominal tuberculosis and peritoneal dialysis (PD)-related condition (3). Detailed history and physical examination are helpful especially in case of secondary SEP (6). The presence of typical radiological signs of SEP can verify the diagnosis preoperatively, hence giving the patient the appropriate management of his condition (6). We present in this report two cases of abdominal cocoons diagnosed preoperatively by abdominal computed tomography (CT) and managed with complete excision of the membrane.

## Case presentation

### Case 1

A 36-years-old male patient from India was presented to our hospital with abdominal pain mainly in the left upper quadrant in association with nausea and vomiting for 1 day. He denied any history of similar attacks before, no personal history of tuberculosis, or contact with any Tb patient. Also, he had no history of previous abdominal surgeries. Upon examination, the patient's vital signs were found stable with his abdominal examination was soft with left upper quadrant tenderness. His basic blood workups were not significant, as well as his inflammatory marker was not elevated.

**Figure 1:**
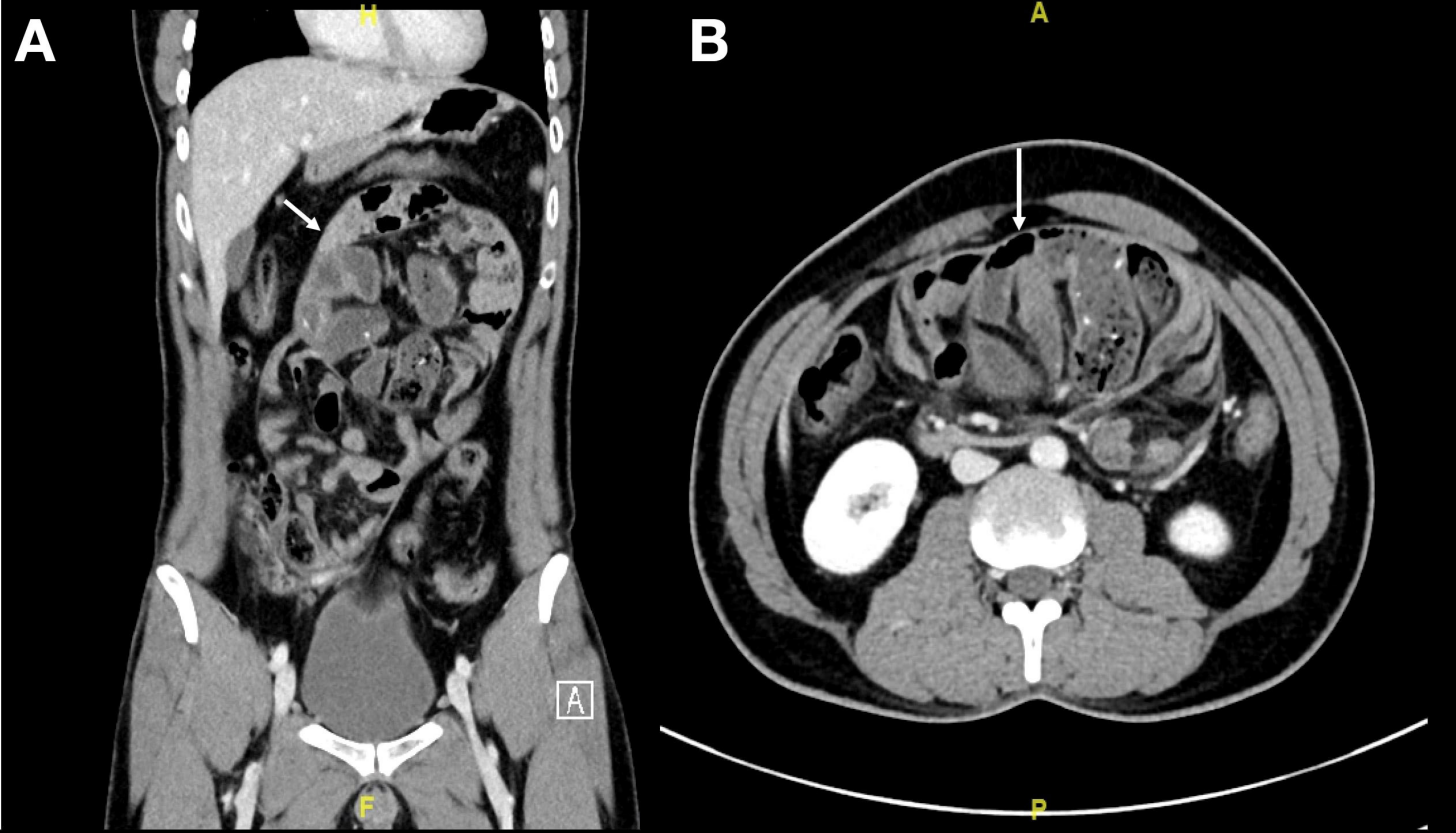
**(A,B)** Abdominal CT scan coronal and axial cut showed small bowel loops clusters at the mid-abdomen and are surrounded by a thick enchanting membrane forming a sac the small bowel loops are arranged radially (Arrow indicates the sac).

Initially, an abdominal X-ray was done for him, which showed non-specific bowel distribution with no air-fluid level. Then, he underwent CT of the abdomen and pelvis with intravenous contrast. It showed a small bowel cluster in the mid-abdomen with a small bowel dilation with a transition zone at the distal ileum, along with a trace amount of stool in the colon. There were no signs of bowel perforation or ischemia. The small bowel was encased by a membrane forming a sac with a small amount of free fluid in it (Figures 1A,B). The finding gives us the impression of sclerosing encapsulating peritonitis.

**Figure 2:**
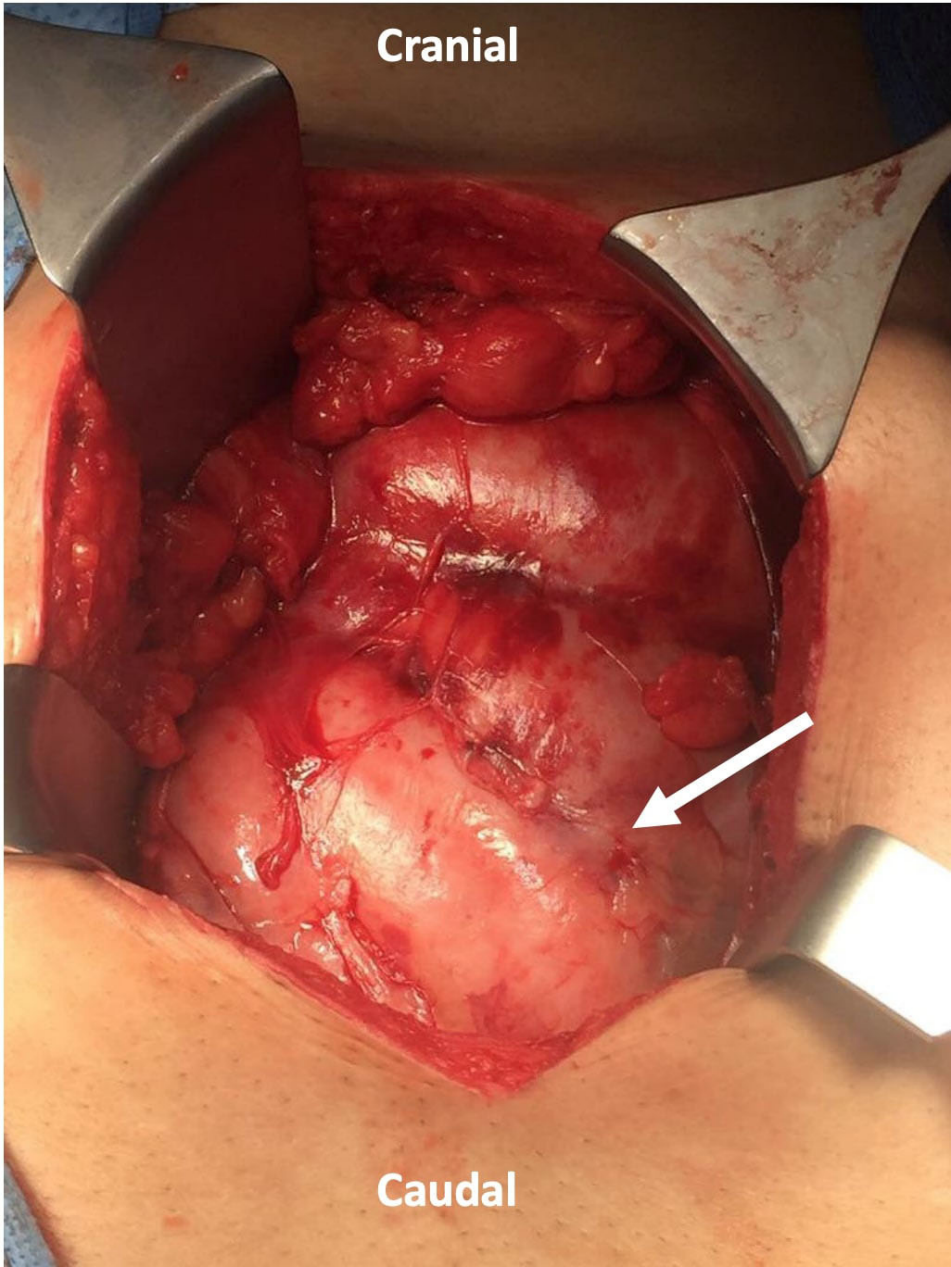
Laparotomy incision showing a thick fibrous membrane encasing the small bowel resembling a cocoon (Arrow indicates the sac).

He underwent exploratory laparotomy with the intraoperative finding of a thick membrane covering the small bowel (Figure 2); adhesiolysis was done and the sac was excised from the small bowel to the root of the mesentery. The sac was sent for pathology review and mycobacterium culture. There was no bowel perforation or gangrene. Also, appendectomy was done for the patient anticipating difficult intraoperative access in case he had appendicitis in the future. His postoperative course was smooth without complication, diet advanced as tolerated, and he was discharged on postoperative day 5.

He was last seen in the clinic 3 months after the surgery; he was symptom free with his wound healed very well. The pathology report showed a fibrocollagenous membrane with chronic lymphocytic inflation. Mycobacterium culture was negative.

### Case 2

A 26-year-old patient from India, not known to have any chronic medical illness, presented to our ER department complaining about generalized abdominal pain for 5 days associated with nausea and vomiting for 1 day. He had a similar episode 5 months ago, which got resolved spontaneously. He denied any personal history of Tb or contact with any Tb patient. Also, he had no history of previous abdominal surgeries. Upon examination, the patient's vital signs were found stable, and his abdominal examination was distended with left- and right-upper quadrant mild tenderness without any sign of peritonitis, and digital rectal examination revealed an empty rectum with no mass. His basic blood workups showed high WBC, reaching 18.8 (k/ul), as well as his inflammatory marker was elevated.

**Figure 3:**
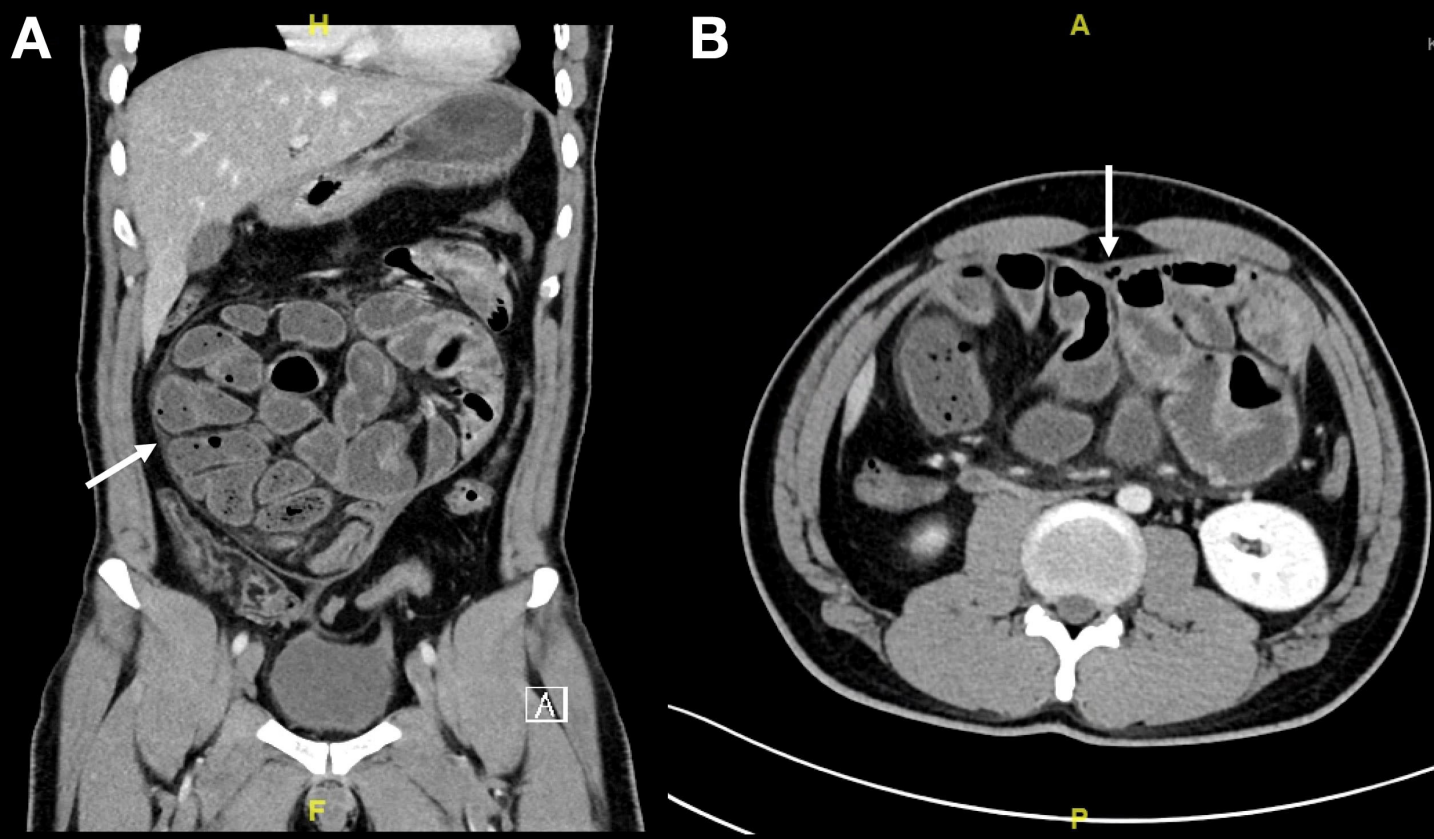
**(A,B)** Abdominal CT scan coronal and axial cut showed multiple dilated fluid-filled small bowel loops involving the jejunum and ilium in the center of the abdominal cavity with thin soft tissue non-enhancing capsule encasing the small bowel loops (Arrow indicates the sac).

Initially, an abdominal X-ray was done for him, which showed non-specific bowel distribution with no air-fluid level. Then, he underwent a CT of the abdomen and pelvis with intravenous contrast. It showed multiple dilated fluid-filled small bowel loops in the center of the abdominal cavity with thin, soft tissue non-enhancing capsule encasing the small bowel loops with mesenteric congestion involving small and large bowel loops with a small amount of free fluid in it (Figure 3). The finding gives us the impression of sclerosing encapsulating peritonitis.

**Figure 4:**
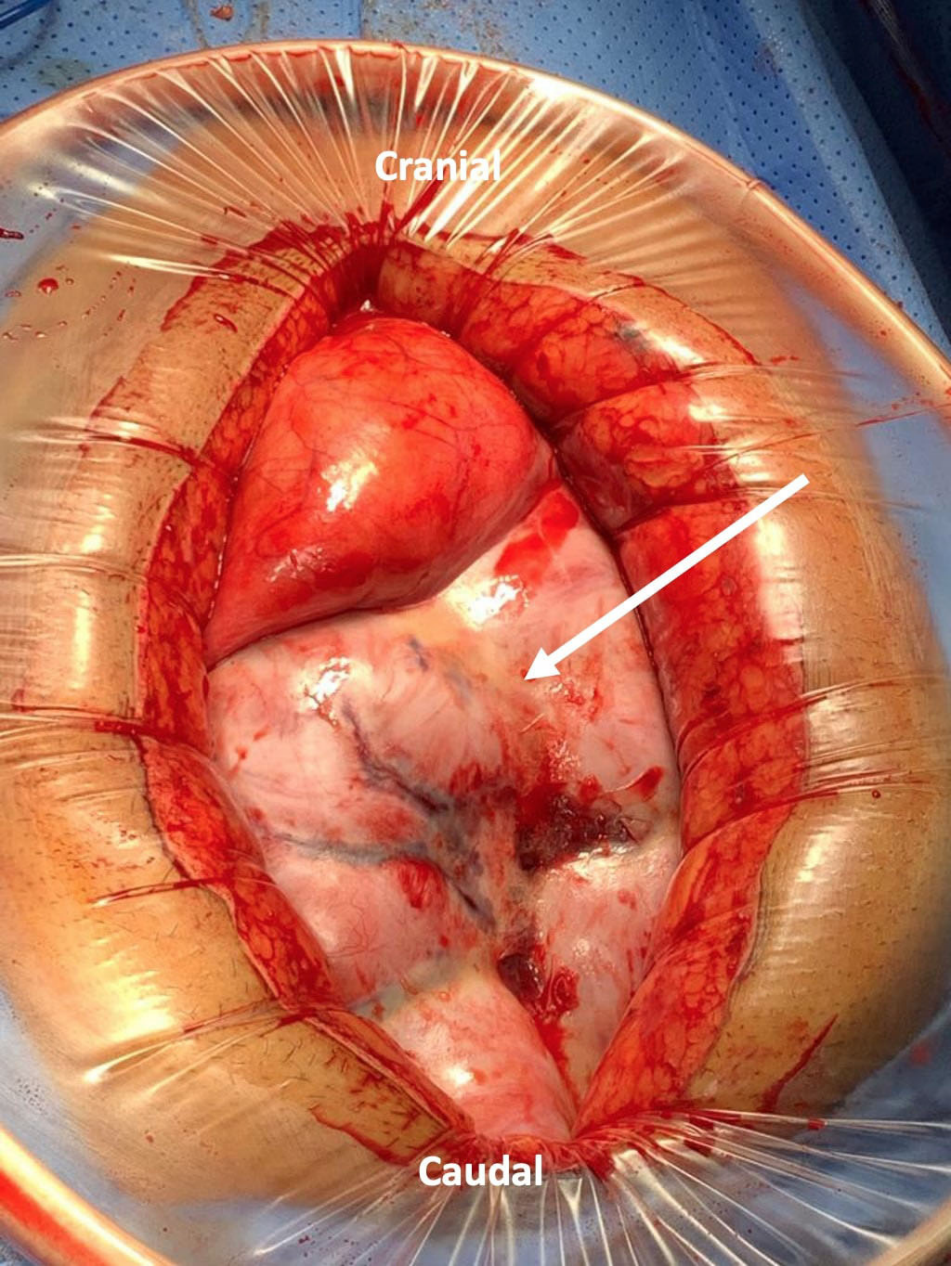
Laparotomy incision with wound protector showing a thick fibrous membrane encasing the small bowel resembling a cocoon (Arrow indicates the sac).

He underwent exploratory laparotomy with the intraoperative finding of thick membrane covering the small bowel (Figure 4); adhesiolysis was done and the sac was excised from the small bowel to the root of the mesentery. The sac was sent for pathology review and mycobacterium culture. There were no bowel perforation or gangrene. His postoperative course was smooth without complication, diet advanced as tolerated, and he was discharged on postoperative day 5.

He was last seen in the clinic 3 months after surgery; he was symptom free with his wound healed very well. The pathology report showed a thick fibrocollagenous membrane with chronic lymphohistocytic infiltrate. Mycobacterium culture was negative.

## Discussion

SEP is a rare cause of intestinal obstruction in which the bowel and internal abdominal organs are encased with a fibrocollagenous membrane (1). There are multiple nomenclatures in the literature, describing various conditions where the gut is encased with a membrane (3). It includes peritoneal encapsulation (PE), idiopathic and secondary SEP, as well as AC (3). PE was first described by Cleland in 1868 (7). It is surrounded by normal peritoneum derived embryologically from the yolk sac peritoneum (8). PE is commonly associated with intestinal or colonic malrotation, cryptorchidism, and hernia (9). It is also accompanied by the absence of omentum and gastrocolic ligaments (9). PE is usually asymptomatic, easily removable, and is not associated with any inflammatory process unlike SEP (8).

Abdominal cocoon terminology was first used by Foo et al. in 1978; however, the condition was initially described by Owtschinnikow in 1907 as peritonitis chronica fibrosa incapsulata (2, 4). SEP is classified further depending on the etiology and pathogenesis into primary or secondary (3). Primary SEP has been named idiopathic SEP or abdominal cocoon syndrome (8). It is usually labeled as primary SEP when there is no cause that can be found to explain this phenomenon after a detailed clinical, radiological, and histopathological assessment (8). Idiopathic SEP has been classically described to occur in young adolescent girls living in tropical areas with the etiology believed to be related to retrograde menstruation or viral gynecological infection *via* the fallopian tubes. In a recent article by Akbulut, he reported on a review of 193 published cases, most of which were from China, India, and Turkey, showing that it is actually twice as common in men than in women (6). These findings are contradicting the theories of retrograde menstruation, leaving the door open for other alternative etiology, such as development disorder from vascular anomalies and omental hypoplasia (6, 10). For the time being, the exact cause of primary SEP remains a mystery to be identified. Unlike secondary SEP, where there is a definitive etiology cause of disease such as peritoneal dialysis, pelvic inflammatory disease, tuberculosis, and sarcoidosis (1, 8). History of abdominal trauma, abdominal surgery, autoimmune disease, peritoneal shunts, and beta-blocker have been reported as a cause of secondary SEP (8). Few reported its association with hepatitis C, liver transplantation, as well as abdominal free gas (9). Table 1 summarizes some of the causes of secondary SEP mentioned in the literature.

**Table 1:** Etiology of secondary SEP.

Intervention or local cause - Peritoneal dialysis. - Intraperitoneal chemotherapy. - Trauma-related. - Liver transplant. - Abdominal surgery. Systemic cases like Infection, Drugs, and Medical disease - Abdominal tuberculosis - Recurrent peritonitis - Granulomatous peritonitis - Beta-blocker - Chemotherapy - Asbestos exposure - Endometriosis - Liver cirrhosis - Gastrointestinal malignancy

Furthermore, SEP can be categorized into three types depending on the extent of the membrane encasement (8). Type 1 is the small bowel that is partially encased by a membrane, type 2 is the whole small bowel that is encased by the membrane, while type 3 is the whole small bowel and other intraperitoneal organs (colon, stomach, liver, and ovaries) which are encased by the membrane (1, 3, 8). Machado et al. reported in their systematic review of 118 patients that 43% were type 1 and 31% were type 2, while only 25% were type 3 (3). Li et al. suggested another way to classify SEP depending on the presence of a second enterocoelia (11). Type 1 is the absence of a second enterocoelia, while type 2 is the presence of a second enterocoelia (11). They also reported in their study involving 26 abdominal cocoon patients that 30% were type 2 having a second enterocoelia, while 70% were type 1 without enterocoelia (11).

Historically, SEP was considered to be more frequent in young girls living in tropical areas, which supported the theory of retrograde menstruation *via* the fallopian tube; however, recent reports including a systematic review showed that it is more frequent in males (12, 13). In our review of the reported cases in the Arabian Gulf region, we found only eight cases of SEP, which are summarized in Table 2. A total of 66 out of 89 patients were male in two large studies reported by Li et al. and Wei et al. with primary SEP, which reduces the support for retrograde menstruation therepy (14, 15). So far, the pathogenesis of primary SEP remains a mystery with multiple theories regarding its pathogenesis; however, none are completely accepted in the literature (16).

**Table 2:** Clinical summary of reported cases in the Arabian Gulf region.

	**Ref**.	**Country**	**Age/sex**	**Clinical presentation**	**Diagnostic**	**Etiology/Histology**	**Treatment**	**Outcome**
1	Al-abassi et al. (17)	United Arab Emirates	25 y/Male	Central abdominal pain for 1 day, with nausea and vomiting	Intraoperative exploratory laparotomy	Not mentioned	Surgery exploratory laparotomy adhesiolysis	Uneventful 3 months follow up
2	Al Saied et al. (18)	Saudi Arabia	24 y/Male	Crampy abdominal pain for 2 days with nausea and vomiting	CT abdomen showing membrane enveloping the small bowel giving appearance of cocoon	Non-specific fibrosis	Surgery laparotomy adhesiolysis, no bowel resection	Uneventful 5 months follow up
3	Jaber et al. (19)	Saudi Arabia	37 y/Male	Blunt abdominal trauma after road traffic accident	CT abdomen showed loculated hematoma within a sac containing small bowel loops	Low cuboidal mesothelial cells composed of congested fibrofatty and collagenous tissue consistent with sclerosing encapsulating peritonitis	Surgery exploratory laparotomy mesenteric tear, excision of the sac, control of the bleeding	Uneventful hospital stay
4	Meshikhes et al. (20)	Saudi Arabia	45 y/Male	6 months history of colicky abdominal pain with nausea, vomiting and weight loss	CT abdomen showing conglomerate of intestinal loops surrounded by thick sac like membrane	Consistent with Sclerosing peritonitis	Surgery laparotomy adhesiolysis and appendectomy	1 year follow up without complication
5	Al Ani et al. (21)	Bahrain	39 y/ Male	Long standing (years) colicky abdominal pain with weight loss	CT abdomen showing clumping of ileal loops with thin capsule surrounding it	Dense poorly cellular collagnise fibrous tissue consistent with sclerosing encapsulating peritonitis	Surgery, laparotomy excision of the whole membrane, adhesiolysis and appendectomy	Uneventful 5 months follow up
6	Al-Azzawi et al. (22)	Oman	44 y/Male	Abdominal pain for 2 days with constipation	CT abdomen showed a sac containing distal jejunum and ileum	Fibrocollagenous tissue	Surgery laparotomy adhesiolysis, no bowel resection	Uneventful 11 days follow up
7	Arif et al. (23)	Iraq	30y/ Male	Abdominal pain, bilious vomiting	Intraoperative exploratory laparotomy	Chronic inflammatory process	Surgery, exploratory laparotomy adhesiolysis, no bowel resection	Discharged home with no complication
8	Arif et al. (23)	Iraq	35 y/ Male	Chronic right lower quadrant abdominal pain	Diagnostic laparoscopy	Chronic inflammatory process	Surgery Diagnostic laparoscopy adhesiolysis, no bowel resection	Discharged home with no complication

Intestinal obstruction is the most common presentation of SEP (13). The most common manifestations of it are abdominal pain 72%, abdominal distension 44%, abdominal mass 30%, and nausea/vomiting as reported by Machado et al. (3) which included a systematic review of 118 patients. They also reported the mean age of presentation was 39 years (3). The presentation is variable and includes acute, subacute, or chronic presentation (13) Hence, the symptoms can range from acute small bowel obstruction with abdominal tenderness to chronic abdominal pain in association with nausea, anorexia, and weight loss (24). Palpable abdominal mass during the examination can be present in the case of a second enterocoelia (11, 24). Weight loss and malnutrition are more pronounced in chronic cases; as Li et al. reported in their study of 65 patient that 75% of AC patients were having a BMI of < 18.5 kg m^2^ (14).

The diagnosis of SEP needs a combination of detailed medical history, full physical examination, a high index of suspicion, and a variety of laboratory and radiological investigations (25). The most common presentation of the symptomatic abdominal cocoon is acute intestinal obstruction through the emergency department (10). Detailed medical and surgical history with physical examination and a variety of laboratory investigations rule out the most common causes of intestinal obstruction, which is reported as 60–80% caused by postoperative adhesion. Only 6% are caused by unusual conditions, such as internal hernia, voluminous intussusception, and chronic idiopathic intestinal pseudo-obstruction (26). Internal hernia is an important differential diagnosis to SEP with a similar CT finding; however, it usually lacks a membrane-like sac in the CT scan (27). Due to chronic idiopathic intestinal pseudo-obstruction, it shows dilation in the small and large bowels with no membrane lacking sac (26). Classically, SEP is diagnosed intraoperatively with a thick membrane encasing the intestine; however, recently, radiological imaging has proven to be helpful in preoperative diagnosis (28). Preoperative diagnosis of SEP can help tailor the surgical planning as well as prevent any unnecessary operative intervention and possible bowel resection (14). Diagnosing SEP requires a combination of thorough patient history and examination to identify any risk factor for secondary causes and tailored radiological workup with a high index of clinical suspicion (14). There are several radiological modalities to further help diagnose SEP, starting with plain abdominal X-ray, oral contrast (barium or gastrografine) studies, ultrasonography, progressing to abdominal CT, which is the most useful modality, and finally on rare occasions contrast-enhanced magnetic resonance imaging (MRI) (3, 26). In patients suspected of having secondary SEP, further investigation is required to confirm the predisposing condition. It includes erythrocyte sedimentation rate, sputum test for tuberculosis, or even diagnostic laparoscopy and biopsies to evaluate inflammatory and malignancy causes (3, 6, 29). Further work would be needed to evaluate other autoimmune and gynecological inflammatory conditions (3, 6).

Usually, the plain abdominal X-ray is the first radiological modality in SEP patients, which might show signs of bowel obstruction, including air-fluid levels, and dilated bowel loops, and scarcely will show peritoneal calcification (3, 13, 30). Oral contrast studies will show conglomerated and accumulated small bowel at the center of the abdomen revealing a sign called cauliflower sign (6). Ultrasounds have been reported in the literature by some authors to facilitate the diagnosis of SEP (3, 26). Sonographic images may reveal dilated small bowel loops, trilaminar appearance of the bowel wall, membrane formation, and tethering of the bowel to the posterior abdominal wall (26). Abdominal CT is one of the highly used sensitive radiological modalities reported in the literature (3, 6). It will show several radiological characteristic signs of SEP like small bowel dilation at the midline encased by a sac or a thick membrane, small amounts of encapsulated effusion in the sac, intestinal obstruction, mesenteric thickening, the greater omentum is hypoplastic or absent, they also mentioned cocoon-like membrane, calcified small intestinal wall, swelled lymph nodes, or mesenteric fat gains (3, 13, 26, 31).

In patients who present with complete bowel obstruction secondary to SEP, the standard approach is exploratory laparotomy with complete membrane excision and adhesiolysis (9, 16, 32, 33). Bowel resection with primary anastomosis or stoma creation is only indicated if the bowel is gangrenous or perforated (15). Machado reported the chance of recurrence in patients who have failed complete or partial excision of the sac (3). Manipulation during surgery needs to be delicate and precise to completely release the neck of the membrane along the duodenojejunal junction and superior mesenteric vessel to avoid iatrogenic bowel or vessel injury (15, 33). Some authors advocate for appendectomy during the same setting to avoid entering a hostile abdomen in the future, especially with atypical presentation (15, 33). The association of SEP with embryologic origin has been suggested in the literature especially in the presence of congenital anomalies, such as the absence of greater omentum and gastrocolic ligament (9). Also the presence of a hernia, accessory sac, and cryptorchidism has been reported associated with SEP and should be evaluated intraoperatively (11). Laparoscopic interventions have been reported in the literature with the advantage of having both diagnostic and therapeutic; however, with increased the risk of iatrogenic bowel injury during trocar insertion (6). In a stable patient with a virgin abdomen presenting with intestinal obstruction, conservative management can be considered (3).

The most common postoperative complications are early postoperative small bowel obstruction (EPSBO), intraabdominal infection, enterocutaneous fistula, short-bowel syndrome, and bowel perforation (6, 8, 14). EPSBO occurred within 30 days of the procedure, and it is caused by excessive manipulation of the bowel, long operative time, intestinal edema, as well as serosal tears (6, 8). Frequently, it is managed conservatively with bowel rest, and total parenteral nutrition (TPN) without requiring surgical reoperation (6, 14). Li et al. suggested in a review of 65 patients with SEP that giving TPN in combination with somatostatin with a low dose of the steroid, if necessary, is a part of their protocol (14). Machado reported in his systematic review that postoperative obstruction occurs in around 5.9% of reported cases in the literature (3). Singh et al. (16) reported in their study that the chance of reoperation was 6.6%.Click or tap here to enter text. The mortality rate for SEP patient in the literature range from 45 to 82% occurring week or months after surgery (3).

## Conclusion

In conclusion, our aim of this report is to raise awareness of this condition typically presenting with intestinal obstruction. Detailed history and physical examination are helpful, especially in the case of secondary SEP. The presence of typical radiological signs of SEP can verify the diagnosis preoperatively, hence giving the patient the appropriate management of his condition. Intraoperative finding of a thick membrane covering the bowel is the golden standard for diagnosis. Most primary SEPs are managed with surgical exploration and excision of the sac; however, conservative management can be considered in some cases of secondary SEP. EPSBO is the most common postoperative complication with most cases managed with bowel rest and TPN. Further study is required to investigate the pathogenesis of SEB as well as the long-term outcome in terms of surgical intervention and conservative medical management.

## Data availability statement

The original contributions presented in the study are included in the article/supplementary material, further inquiries can be directed to the corresponding author/s.

## Ethics statement

Ethical review and approval was not required for the study on human participants in accordance with the local legislation and institutional requirements. Written informed consent was obtained from the patients. Written informed consent was obtained from the individual for the publication of any potentially identifiable images or data included in this article.

## Author contributions

HAA, AA, MA, HMA, and SA: conception and design, review of the literature, and critical revision of the manuscript and factual content. All authors contributed to the article and approved the submitted version.

## Conflict of interest

The authors declare that the research was conducted in the absence of any commercial or financial relationships that could be construed as a potential conflict of interest.

## Publisher's note

All claims expressed in this article are solely those of the authors and do not necessarily represent those of their affiliated organizations, or those of the publisher, the editors and the reviewers. Any product that may be evaluated in this article, or claim that may be made by its manufacturer, is not guaranteed or endorsed by the publisher.

## Abbreviations

SEP: Sclerosing encapsulating peritonitis
PD: peritoneal dialysis
PE: peritoneal encapsulation
AC: abdominal cocoon
CT: computed tomography
MRI: magnetic resonance imaging
EPSBO: early postoperative small bowel obstruction
TPN: total parenteral nutrition.
